# Problem Gambling among Young People in Sub-Saharan Africa

**DOI:** 10.3389/fpubh.2018.00023

**Published:** 2018-02-09

**Authors:** Derrick Ssewanyana, Byron Bitanihirwe

**Affiliations:** ^1^Centre for Geographic Medicine Research Coast, Kenya Medical Research Institute (KEMRI), Kilifi, Kenya; ^2^Utrecht Centre for Child and Adolescent Studies, Utrecht University, Utrecht, Netherlands; ^3^Center for Public Health Initiatives, University of Pennsylvania, Philadelphia, PA, United States

**Keywords:** addiction, gambling, intervention, public health, youth

## Abstract

Gambling is a cross-cultural and global activity which typically involves the wagering of money or an item of monetary value on an outcome that is governed by chance. Although gambling is positioned as a legitimate recreational and leisure activity within sub-Saharan Africa (SSA), there is widespread recognition among healthcare professionals and policy-makers that gambling has the capacity to become dysfunctional in a minority. Emerging knowledge suggests that problem gambling is rapidly evolving in to a public health concern in SSA, especially among youth. This article focuses on problem gambling among young people in SSA with an emphasis on three key themes: (1) gambling behavior and patterns in SSA; (2) public health and socioeconomic implications of gambling in SSA; and (3) public health policies and interventions for addressing this issue. We believe that collaborative efforts between government, prevention specialists, legislators, researchers, treatment providers, and other stake holders can influence the uptake of research findings necessary to implement social policies and design effective public health intervention options to combat problem gambling and its associated implications among young people in SSA.

## Introduction

Over the recent years, many parts of the developing world have experienced unprecedented increases in gambling availability, participation, and expenditure. This pattern of growth has been evident in regions such as sub-Saharan Africa (SSA) (1, 2). Indeed, legal casinos are currently known to operate in a number of countries in SSA, including Angola, Botswana, Democratic Republic of Congo, Gabon, Gambia, Kenya, Ghana, Lesotho, Liberia, Namibia, Nigeria, Rwanda, Senegal, South Africa, Swaziland, Tanzania, Uganda, and Zimbabwe. South Africa currently holds the most casinos on the continent with 38 legally operating casinos (3). While the gambling industry is considered to have a beneficial impact on the economy through employment and taxation (1), unfortunately, gambling addiction is increasingly evolving into a public health concern in SSA, especially among young people (aged 10–24 years old).

Gambling addiction has been defined as a biopsychosocial disorder characterized by a persistent and recurrent maladaptive pattern of gambling behavior (4). This disorder is associated with adverse psychological, physical, economic, social, and legal outcomes (5, 6). Initially included in the Diagnostic and Statistical Manual of Mental Disorders (DSM)-III under the name “pathological gambling” (7), this condition was subsequently renamed “gambling disorder” (GD) in the DSM-V and classified alongside substance-related disorders (8, 9). Indeed, the clinical literature points toward high rates of comorbidities between GD and substance-related disorders, their overlapping neural circuitry (4, 10, 11), shared clinical presentation (i.e., cognitive features) and genetic predisposition (12–18) in addition to similarities between their treatment methods (11).

As opposed to the burgeoning literature exploring gambling and GD in Western nations (19, 20), there is a paucity of research on gambling behavior (e.g., frequency and amount of gambling, forms of gambling, and gambling motivations), the prevalence of GD, and identifying high-risk groups for developing GD, such as adolescents, in SSA. This information gap is important in terms of gaining a better understanding and knowledge of these variables and will assist in designing effective preventive and treatment programs. In this context, the present article aims to provide a fresh perspective on the emerging issue of problem gambling among young people in SSA.

## Gambling among Young People in SSA

With the rapid growth of the gambling industry in many parts of SSA, such as Nigeria, South Africa, and Kenya, which at times is coupled with weak regulatory environments (1), young people are increasingly exposed to gambling practices. A recent survey evaluating gambling-related activities in 3,879 youth aged between 17 and 35 (based on the African Youth Charter) in Kenya, Uganda, South Africa, Ghana, Nigeria, and Tanzania found that 54% of youth in SSA have engaged in some form of gambling activity (2). In this study, Kenya was reported as the country with the highest number of youth who had previously participated in gambling or betting at 76% followed by Uganda at 57% while Ghana had the lowest number at 42% (2). Due to their high propensity for risk taking (21), and not being aware of the potential undesirable effects of such behaviors (e.g., poor physical health, disrupted familial/peer relationships, increased risk of crime, poor academic performance, and an increased risk of mood disorders and suicide) (22, 23), young people’s engagement in gambling recreation may culminate in GD.

Sub-Saharan Africa is home to the world’s largest youthful population (24) with eight of the world’s top 10 countries with the youngest populations currently being in SSA and by 2050 the region will be home to all 10 (25) yet only a handful of studies exist on GD and their associated problems among young people (Table 1).

**Table 1:** A list of studies focusing on gambling-related activities and GD in SSA.

Reference	Country/region	Database	Gambling prevalence	Prevalence of GD	Age group (year)
Enwereuzor et al. (26)	Nigeria	NLM/Pubmed	Not reported	Not reported	16–34
Heap (27)	Nigeria	NLM/Pubmed	Not reported	Not reported	12–14
Abdi et al. (28)	Ethiopia	NLM/Pubmed	Not reported	6.9%	12–21
Skaal et al. (29)	South Africa	NLM/Pubmed	Not reported	28.3%	>18
Sinclair et al. (30)	South Africa	NLM/Pubmed	Not reported	Not reported	>18
Sinclair et al. (31)	South Africa	NLM/Pubmed	Not reported	Not reported	18–72
Sharp et al. (32)	South Africa	NLM/Pubmed	Not reported	10.8%	>18
Scott and Barr (33)	South Africa	NLM/Pubmed	Not reported	Not reported	21–60
Dellis et al. (34)	South Africa	NLM/Pubmed	Not reported	Not reported	18–81
Peltzer and Thole (35)	South Africa	NLM/Pubmed	Not reported	Not reported	18–32
Langa (36)	South Africa	NLM/Pubmed	Not reported	Not reported	13–18
Kincaid et al. (37)	South Africa	NLM/Pubmed	Not reported	3.2%	>18
Rataemane and Ligthelm (38)	South Africa	PsychInfo	Not reported	Not reported	>18
Collins and Barr (39)	South Africa	Google Search	Not reported	4.8%	>18
Collins and Barr (40)	South Africa	Google Search	Not reported	4.6%	>18
Collins and Barr (41)	South Africa	Google Search	Not reported	4.8%	>18
Ahaibwe et al. (42)	Uganda	Google Search	24.3%	Not reported	>18
GeoPoll (2)	SSA	Google Search	42–76%	Not reported	17–35

*GD, gambling disorder; SSA, sub-Saharan Africa; NLM, The National Library of Medicine*.

Importantly, some of these studies point to a significant burden of gambling problems among young people in SSA (28, 39–41). For example, one study conducted among high school adolescents in Ethiopia reported that 73% of the adolescents had ever gambled. Of these, 37% were at risk for severe problematic gambling while 7% were already problematic or pathological gamblers as screened by the DSM-IV-Juvenile checklist (28). Most studies from the region concur that young males face a higher risk for GD (28, 32), however, young females’ increasing involvement especially in “closet” forms of gambling, including mobile internet-based lotteries and games (42) such as “Fahfee”—which is widely played by black South African women living in townships—has been documented (33, 43).

Gambling in SSA takes on various forms, including commercially legalized gambling options such as casinos, pool games, bingo, sports betting, scratch cards and lotteries. Studies indicate a high cultural variation in local gambling options, accessibility, and participation (28, 33, 35, 36). For instance, in South Africa it has been reported that people in townships are more likely to participate in gambling activities involving dice and cards that are perceived to be “fairer” as opposed to lottery and casino based activities that are seen to be “rigged” and unfair (33). This can make gambling readily accessible to those without adequate financial resources, such as minors and the urban poor. It follows that different gambling practices have been reported across socioeconomic strata and also between men and women (boys and girls). Indeed, a study conducted in three South African townships found a disproportionately higher prevalence (7%) of gambling-related problems in poor households compared to affluent households (3%) (44).

In considering these points, several models have been proposed to explain the acquisition of gambling behaviors among adolescents and young people such as social learning theory (45) with evidence suggesting that adolescents may engage in more risky behavior in the presence of peers (i.e., peer-to-peer learning) (46). In this regard, a recent survey conducted in Uganda found that 39% of respondents reported that they were aware of minors who engaged in gambling activities (42). Besides the influence of peers and aggressive marketing efforts from the gambling sector (42), GD and associated problems sometimes trace their roots in a family setting where young people are introduced to gambling at a young age (34). In a study conducted in South Africa 13% of the gamblers had experienced gambling problems in their families while they were younger (i.e., before young adulthood) (34). Indeed, in various settings, young people perceive gambling as an acceptable activity to the extent that some consider it an alternative source of livelihood toward which they would rather devote substantial time and energies (27, 42).

## Implications of Gambling

The gambling industry has established itself as a prominent social and economic force with significant impact on job creation and revenue generation (1, 44). It follows that some young people have been reported to hold positive attitudes toward rewards from gambling, such as entertainment, relaxation, masculinity, and financial benefits (36, 42, 44). Whether such rewards actually materialize remains questionable. Gambling has, however, been shown to have far-reaching negative implications at individual, family, community, and societal level; most of which remain insufficiently explored within the SSA setting. At the individual level, compulsive gambling problems affect a measurable proportion of young gamblers (28) and may manifest in both psychiatric (e.g., anxiety, depression, and sleep deprivation) (28, 32, 36) as well as long-term physical conditions (e.g., cardiovascular disease, peptic ulcer disease, and hypertension) (22). Surprisingly, very few gamblers would admit that gambling is an addictive habit (44).

Unfortunately, a diagnosis of GD is often missed in clinical or population settings implying that those in need of medical and social care often fail to access it when most needed. Minors and young people who indulge in gambling are found to perform poorly in their studies, to lose their school/tuition fees in gambling-related activities, and to engage in risky behavior, such as alcohol use and high-risk sexual behavior (28, 42). Financial hardship is another cross-cutting concern. For instance, in South Africa, gamblers recounted experiences of landing themselves in debt, family financial hardships, and high levels of poverty in their communities (44). As with regular gamblers in Uganda, South African gamblers reported that they spend an equivalent of 12–25% of their salary on gambling-related activities (34, 42). Almost 63% of them agreed that gambling does not have any positive impact on their household welfare (42). Woefully, it is mainly the poorest strata of these societies who spend most of their income on gambling (34, 47), as such a vicious cycle of poverty ensues. Other notable impacts of gambling include loss of productive school or work hours, truancy, and domestic and community violence as well as loss of family assets (42, 44).

## Interventions and Social Policy for Gambling among Young People in SSA

Treatment of GD remains a significant challenge with the most effective treatments, including a varied combination of psychotherapy, pharmacotherapy, financial education, and self-help interventions. Unfortunately, it appears that the pervasive stigma surrounding GD may prevent people from seeking treatment. In this context, the first step of the treatment process typically involves convincing the patient that “*most people can gamble successfully without committing a moral transgression*” (48).

Despite the surge in research from developed countries targeting “druggable” neural targets/systems linked to GD such as opioid antagonists, dopamine agonists and glutamatergic agents, small sample sizes, non-representative groups (e.g., those without co-occurring psychiatric diagnosis), and varied response measures have been reported to differ among published studies (49, 50). These factors make it difficult to assess the effectiveness of pharmacotherapy for GD. It is, however, noteworthy that medication appears to work more effectively when used in conjunction with psychological treatment. In this respect, the use of cost-effective psychological interventions, such as self-directed interventions (e.g., Gamblers Aware), professionally delivered treatment (e.g., cognitive-behavioral therapy), as well as brief and motivational therapy—which focus on changing the maladaptive thinking processes linked to GD—have shown efficacy in the treatment of GD (51). Similarly, addressing problems associated with the gambling industry, such as addiction and underage gambling, have revealed promising results for tackling this issue (52). The provision of low-cost interventions designed to prevent people shifting from being “at-risk” of developing GD to developing a full disorder may prove helpful when working with patients who are unwilling to seek formal or more intensive treatment for their disorder (52). Other cost-effective methods to tackle GD include responsible gambling programs and educational initiatives (52). Responsible gambling programs provide information and education about the risks of gambling and counseling support to gamblers who are experiencing problems with gambling. In contrast, educational initiatives consist of educational and informational/awareness campaigns to prevent problem gambling. Because adolescents may be especially vulnerable to the harmful effects of gambling they represent a population of interest in terms of these programs and initiatives (52).

As the gambling industry in SSA continues to flourish, the implementation of regulations and formal laws that govern this expansion and assess the impact of gambling upon society will represent key policy initiatives. Policies that enforce that gambling establishments are not situated close to one another (i.e., outlet density) will be important and have already been pushed for in several SSA countries, including Uganda, Tanzania, and South Africa. As another public policy initiative, self-exclusion programs—as seen in South Africa—represent a legislated process allowing for a person to exclude themselves from gambling activities (30). Furthermore, laws preventing gambling centers from setting up shop close to schools and universities in addition to enforcing a limitation of opening hours and tighter restrictions on entry and the advertising of gambling establishments will also be of significance. However, it is necessary to emphasize that such initiatives will only prove effective if there is widespread adherence and enforcement of these policies and statutes. With this in mind, the potential lack of awareness among retailers and people working in gambling establishments regarding penalties and the respective laws, and among the general public on the serious consequences of GD may in part account as to why underage youth can access gambling opportunities in spite of legal age restrictions (34). Therefore, the respective national gambling boards/commissions should enforce stricter rules as a means of safeguarding minors from engaging in gambling activities. In order to tackle this problem, the development of policies and programs offering information to gambling establishments and retailers regarding the importance of enforcing legal age and legal liabilities, all of which would serve as barriers to underage gambling would be necessary. The introduction of programs with a focus on promoting responsible gambling and availing counseling services (e.g., the National Responsible Gambling Programme in South Africa (23, 37, 52) and the East Africa Centre for Addiction Services in Uganda) has proven instrumental in terms of addressing the issue of GD.

Supportive environments created through solid political measures are effective in changing behavioral patterns (38, 53). As noted previously, the age of exposure and onset of gambling behavior has been strongly associated with GD, with a younger age of exposure or initiation being linked to a greater risk of developing a gambling-related problem (34). Hence, it follows that increasing the age of first exposure to gambling participation by limiting the availability and accessibility to gambling venues, activities, and products, and raising the minimum legal age for gambling are all key regulatory policy development issues. However, in order for any such education program to prove effective, it is vital that policies are adopted that can cultivate environments supportive of behavior change.

Other policy initiatives that should be taken into consideration include those that contribute to the prevention of GD through funding commitments to the implementation and institutionalization of prevention practices (52). Notable examples of programmatic policies include training of health services professionals, community development and education, development of resources for treatment and prevention, and industry education programs targeting venue operators and retailers, all of which should focus on establishing supportive environments as well as enhancing the skills of individuals (52).

## Conclusion and Future Directives

With the expansion of the gambling industry in SSA, opportunities to engage in gambling are numerous. Given the high level of youth unemployment and the genuinely low wages in SSA (54, 55), an increasing number of youth find themselves participating in gambling-related activities without being aware of the potential undesirable effects that may culminate from gambling addiction. Unfortunately, there is a dearth of prevention programs or specific treatment facilities for youth gambling in SSA (37). Therefore, a specific set of actions should be taken. First, rigorous enforcement of laws prohibiting underage gambling should be enacted (42, 52). Second, strategies to increase education and public awareness regarding the issue of problem gambling are of core necessity (52). Third, more resources and funding to help increase the currently scarce mental health and addiction programs related to problem gambling in SSA are needed (56). Fourth, collaborative efforts between governments, private, and civil society sectors as well as prevention specialists, legislators, researchers, and treatment providers will help contribute to the development of social policies and effective public health intervention options for treatment of youth and adults alike with problem gambling within the region. With this in mind, future studies will need to investigate whether there are differences in gambling and problem gambling behaviors, as well as gambling correlates, among people from different SSA countries. This is important given the large cultural diversity that exists in SSA and the distinct gambling culture in each country.

## Author Contributions

Both authors substantially contributed to the conception and drafting of this manuscript.

## Conflict of Interest Statement

The authors declare that the research was conducted in the absence of any commercial or financial relationships that could be construed as a potential conflict of interest. The reviewer SR and the handling Editor declared their shared affiliation.
